# Supplementary material for: The Small RNA Universe of Capitella teleta
Source: Front Mol Biosci. 2022 Feb 25;9:802814. doi: 10.3389/fmolb.2022.802814 (PMC8915122; doi:10.3389/fmolb.2022.802814)
Supplement: Supplementary file 1 [file DataSheet1.ZIP › Supplement/known/CAPTEscaffold_122_9853.pdf]

Provisional ID	:	CAPTEscaffold_122_9853
Score total	:	970.2
Score for star read(s)	:	3.9
Score for read counts	:	960.1
Score for mfe	:	1.6
Score for randfold	:	1.6
Score for cons. seed	:	3
Total read count	:	1895
Mature read count	:	1887
Loop read count	:	0
Star read count	:	8

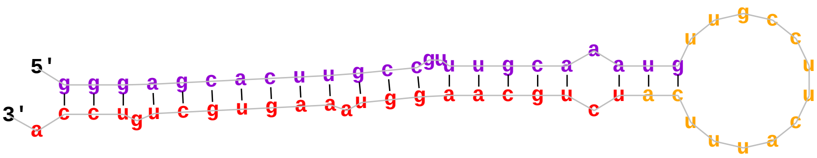

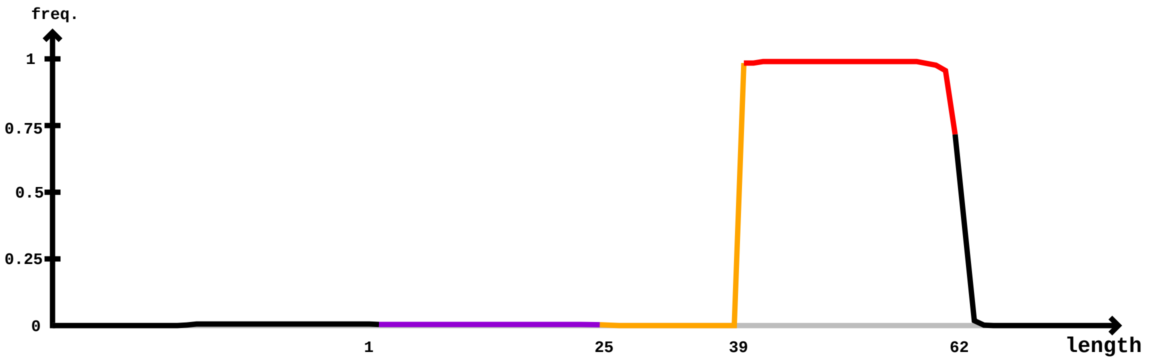

Star Mature

```
5'- ugauaauugcaaauaacaugcgcuuugagguugggagcacuugccguuugcaaauguugccuucauuucaucugcaagguaaagugcuguccaacuugauagugcgaac   -3'    obs
    ugauaauugcaaauaacaugcgcuuugagguugggagcacuugccguuugcaaauguugccuucauuucaucugcaagguaaagugcuguccaacuugauagugcgaac          exp
    ugauaauugcaaauaacaugcgcuuugagguugggagcacuugccguuugcaaauguugccuucauuucaucugcaagguaaagugcuguccaacuugauagugcgaac          known
    ...................(((((.(.(((((((((((((((((..(((((.(((.............))).)))))))).))))))).)))))))).).)))))....    reads    mm    sample
    ............auaacaugcgcuuugagguu.............................................................................    4        0     seq
    .............uaacaugcgcuuugagguu.............................................................................    7        0     seq
    ................................gggagcacuugccguuugcaaa.......................................................    1        0     seq
    ................................gggagcacuugccguuugcaaau......................................................    1        0     seq
    ................................gggagcacuugccguuugcaaaug.....................................................    6        0     seq
    ......................................................................ucugcaagguaaagugcug....................    13       0     seq
    ......................................................................ucugcaagguaaagugcugu...................    13       0     seq
    ......................................................................Acugcaagguaaagugcuguc..................    1        1     seq
    ......................................................................ucugcaagguaaagugcuguc..................    38       0     seq
    ......................................................................ucAgcaagguaaagugcugucc.................    3        1     seq
    ......................................................................ucugcaGgguaaagugcugucc.................    1        1     seq
    ......................................................................Acugcaagguaaagugcugucc.................    2        1     seq
    ......................................................................ucugcaagguaaagugcugucc.................    449      0     seq
    ......................................................................ucugcaagguaaagugcugucU.................    1        1     seq
    ......................................................................Gcugcaagguaaagugcugucca................    1        1     seq
    ......................................................................ucugcaagguaaagugcGgucca................    1        1     seq
    ......................................................................ucugcaagguaaagugcugAcca................    1        1     seq
    ......................................................................ucugcaagguaaagAgcugucca................    1        1     seq
    ......................................................................ucugcaagguaaagugcugucAa................    2        1     seq
    ......................................................................ucugcaagguaaGgugcugucca................    2        1     seq
    ......................................................................ucAgcaagguaaagugcugucca................    2        1     seq
    ......................................................................ucuUcaagguaaagugcugucca................    1        1     seq
    ......................................................................ucugcaagguaaagugcugCcca................    2        1     seq
    ......................................................................Acugcaagguaaagugcugucca................    1        1     seq
    ......................................................................ucugcaagguaaagugcuguAca................    2        1     seq
    ......................................................................ucugcaagguaaagugUugucca................    2        1     seq
    ......................................................................ucugcaagguaaagugcuguccU................    28       1     seq
    ......................................................................ucugcaagguaaagugcugucUa................    1        1     seq
    ......................................................................Ncugcaagguaaagugcugucca................    1        1     seq
    ......................................................................ucugcaaggAaaagugcugucca................    1        1     seq
    ......................................................................ucugcaagguaaagugcugucca................    1012     0     seq
    ......................................................................ucugcaagguGaagugcugucca................    1        1     seq
```

```
                                Star                                 Mature
ugauaauugcaaauaacaugcgcuuugagguugggagcacuugccguuugcaaauguugccuucauuucaucugcaagguaaagugcuguccaacuugauagugcgaac
......................................................................ucugcaagAuaaagugcugucca................   1     1     seq
......................................................................ucugcaagguaaagugcuguccaU...............   126   1     seq
......................................................................ucugcaagguaaagugcugucUaa...............   1     1     seq
......................................................................ucugcaagguaaagugcuguUcaa...............   1     1     seq
......................................................................ucugcaagguaaagugcuguccaC...............   16    1     seq
......................................................................ucugcaagguaaagugcuguccaa...............   121   0     seq
......................................................................ucugcaagguaaagugcuguccaaU..............   12    1     seq
......................................................................ucugcaagguaaagugcuguccaaA..............   7     1     seq
......................................................................ucugcaagguaaagugcuguccaUc..............   4     1     seq
......................................................................ucugcaagguaaagugcuguccaac..............   1     0     seq
......................................................................ucugcaagguaaagugcuguccaaUu.............   3     1     seq
........................................................................ugcaagguaaagugcugucca................   2     0     seq
........................................................................ugcaagguaaagugcuguccaU...............   1     1     seq
........................................................................ugcaagguaaagugcuguccaac..............   8     0     seq
```
